# The quality of work life and turnover intentions among Malaysian nurses: the mediating role of organizational commitment

**DOI:** 10.1186/s42506-020-00048-9

**Published:** 2020-08-14

**Authors:** Luma Ghazi Ibrahim Alzamel, Khatijah Lim Abdullah, Mei Chan Chong, Yan Piaw Chua

**Affiliations:** 1 Department of Nursing Science, Faculty of Medicine, University of Malaya, Kuala Lumpur, Malaysia; 2 Institute of Educational Leadership & Unit for the Enhancement of Academic Performance, University of Malaya, Kuala Lumpur, Malaysia

**Keywords:** Quality of work life, Turnover intention, Organizational commitment, Malaysian nurse

## Abstract

**Background:**

Understanding the factors influencing nurses’ turnover intention, particularly the work life quality and commitment to organization, is important to all countries suffering from nursing shortage. The study aims to determine the mediating role of commitment to organization on work life quality and its relationship with turnover intention among Malaysian nurses.

**Methods:**

A descriptive cross-sectional design, using a self-report survey was conducted on 430 nurses from a teaching hospital from February to April 2019. A structural equation model version 3 was used for testing study hypotheses.

**Results:**

The mediating effect (indirect effect) of organizational commitment on the relationship between work life quality and turnover intention (QWL→OC→IT) was negative with path coefficient − 0.234, whereas the direct effect of work life quality on turnover intention (QWL→IT) was negative with smaller path coefficient − 0.228. This means that the relationship between work life quality and turnover intention was partially mediated by the organizational commitment (*P* < 0.001).

**Conclusion:**

Organizational commitment has a negative partial mediating effect between work life quality of nurses and intention of turnover in teaching hospitals where the organizational commitment significantly reduced the nurses’ intention to leave. The study findings can guide nursing managers to be carefully attended to the levels of nurses’ commitment to their organization.

## Introduction

A wide range of turnover studies indicated serious and complex issues that would affect the employee’s performance and the organization’s services [1]. Many reasons were mentioned in previous studies about turnover such as dissatisfaction, low income, low organizational commitment, and leader’s behavior. The nursing shortage is a big challenge to the industry as it can lead to turnover. It has been identified as a critical problem faced by public hospitals in Malaysia [2]. According to the Malaysian Health Ministry report 2016, there was a shortage of nurses. Hence, managers should understand the reasons for turnover and factors contributing to turnover intention [3].

The turnover of nurses results in an increased cost for the institution to recruit qualified nurses, a decreased patient care quality, and the need to balance between actual and realistic needs [4]. It is necessary to consider not only the organizational needs and desires, but also high-quality working conditions when dealing with keeping good and qualified employees. One of these factors is work life quality which includes any improvement in the organizational culture that supports the growth and development of employees in the organization [4].

Quality of work life is a subjective phenomenon that is impacted by personal perceptions and feelings [5]. It refers to attitude towards the job and general satisfaction with work life and feelings of being valued and respected within the organization [6]. Positive working environment and fulfillment of staff needs will lead to retain the current employees and improve the performance of the organization [4]. Therefore, the organizations should realize “what is required” to retain qualified nurses and to create and maintain suitable working environment that supports competent performance of nursing care [7]. A Taiwanese study found that the work life quality is predictor to nurses’ turnover intention [8]. Previous literature reported that the work life quality had a reverse correlation with turnover intention, which means, high work life quality is necessary to retain employees in their organization [6, 9–11].

Organizational commitment is defined as loyalties to the values and goals of the organization, sense of belonging, dependency, and moral commitment to remain in their organization. The organizational commitment is influenced by work life quality. When the employees are satisfied with work life, they will be more obligated and stay in their organization [4]. Ghoddoosi-Nejad et al. reported that the individual’s work life quality had a direct effect on organizational commitment [12]. A recent study done by Hashempour et al. argued that the work life quality has a positive correlation with commitment to organization [4]. These results were consistent with previous studies confirming that by increasing quality of work life (QWL), the commitment to organization will increase [13–15]. In addition, the studies performed by Ahmad et al. and Lee et al. mentioned that organizational commitment has a reverse correlation with turnover intention [8, 16]. This means that the commitment of employees to their organization by satisfaction and accepting working environment conditions (job demands) will decrease turnover intention. Therefore, organizational commitment is an important predictor of intention to leave; employees who showed high commitment and attachment to their organization were less likely to have intentions to leave and spend longer tenures in same organization [17].

### The purpose and study hypothesis

**Fig. 1:**
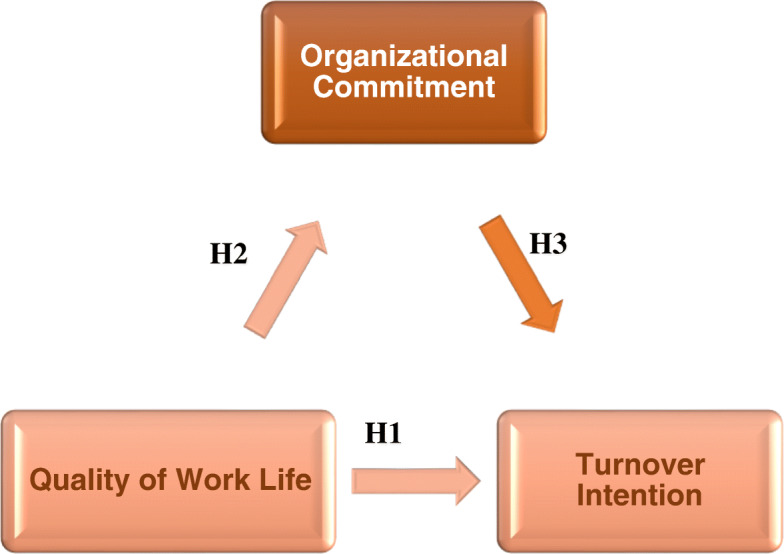
The model clarifying the hypothesis of study

The previous studies clarified that the quality of work life had negative correlation with turnover intention and, positive correlation with organizational commitment [8, 9, 13, 15, 17]. Work life quality covered the satisfaction of employee about job aspects. In addition, an emotional factor was being behind intention to leave [17]. Therefore, this study hypothesized that better work life quality means emotional attachment which helps to decrease nurse’s turnover intention. For that, the present study suggested that as nurses are more satisfied in quality of work life such as work environment (context), work home life balance, work load (design), and social influence (work world), they are unlikely to leave their organization through commitment to it. This study examined work life quality and its relation with intention to turnover, mediated by organizational commitment which might contribute to understanding the strategies of the organization to retain valuable workforce (nurses) by decreasing their turnover intention. The model discussed the direct effect of quality of work life (independent variable) on turnover intention (dependent variable) and the indirect effect (mediating variable) of organizational commitment on the relationship between quality of work life and turnover intention among Malaysian nurses. The study findings provide nursing managers with the information on factors that impact turnover intention, which will facilitate appropriate strategies to retain nurses and decrease this negative phenomenon. Figure 1 introduces the model of study based on the conceptual framework.

## Participants and methods

### Study design and setting

A descriptive cross-sectional design using a quantitative self-report survey was adopted in this study. Stratified sampling technique was conducted among inpatient nurses in the medical, surgical, and special wards/units in a teaching hospital in Malaysia. This government-funded medical institution is located in southwest corner in Malaysia with 1643 beds which are distributed to 44 wards.

### Participants and sample size

The sample size was estimated according to the empirical power tables [18]. It is reference for defining the sample sizes necessary for adequate power in single-mediator models. Fritz and MacKinnon used the following equation to compute power for regression coefficients:

$$ n=\frac{L}{f^2}+k+\mathrm{l} $$

This regression equation consists of sample size (*n*), predictors numbers (*k*), and an effect size (*f*) which measure the ordinary least squares regression, the regression coefficients are used (i.e., 0.14, 0.26, 0.39, and 0.59), and finally, a specific power value (*L*). In this study, the researcher hypothesize that organizational commitment has partial mediator so, τ′ (indirect effect) will be more than zero to achieve 0.80 power in the size α and & β high-small (*α* = 0.26, *β* = 0.14, *τ*′ = 0.39). The sample size was estimated at 405 nurses [18]. Due to variation in the specified population of nurses in different wards or units, stratified sampling technique was preferred to ensure representativeness. The population (nurses) was divided into strata (wards or units in hospital) and the target population was selected by simple random sampling. The size of each stratum was specified using proportional allocation method, where 36% (*n* = 146) of the nurses were selected from the medical wards, 33% (*n* = 134) from critical units, and 31% (*n* = 125) from surgical wards. To achieve 80% power to detect a medium population effect size and two-sided alpha of 0.05 we chose to recruit more than the minimum number of participants needed to achieve a good response rate [19], and possibly missing data. The total number of distributed survey questionnaires was 520. The completed and returned questionnaires were 455. However, twenty-five questionnaires were uncompleted, hence excluded, leaving 430.

### Study instrument

The self-reported questionnaire consists of four sections. The first section collects nine aspects of demographic data such as age, gender, marital status, number of children, educational level, years of experience, ethnicity, the ward which one works in, and income. The second section consists of 42 items from the quality of nursing work life questionnaire (QNWL) [20]. A responding scale ranging from “1 = strongly dissatisfied” to “6 = strongly satisfied” was used for measuring. A low score means a low work life quality. The third section consists of the anticipated turnover scale (ATS) with 12 items measured. It is used to measure the intention of nurses to leave. A total score of less than 42 indicates lesser intention to leave, whereas a total score of more than 42 indicates more intention to leave [21]. The fourth section consists of the organizational commitment questionnaire (OCQ) with 15 items measured. A total score lower than 60 indicates a low commitment to the organization and a total score higher than 91.5 indicates a higher commitment to the organization. However, a mean score between the 60 and 91.5 indicates moderate commitment [22]. A responding scale ranging from “1 = strongly disagree” to “7 = strongly agree” was used in both ATS and OCQ.

The questionnaire was translated from English to Bahasa Malaysia, and then back translated to English by bilingual professional translator. The questionnaire in both languages was reviewed by four nurse academicians. Little modifications were done according to their recommendation for the clarity and accuracy of the questionnaire.

Content and face validity were conducted by two experts academicians from university and two experts from health profession according to checklist for Department of Nursing Science in Faculty of Medicine University of Malaya. Experts were asked to evaluate the overall validity of all the instruments on four areas pertaining to its relevance, clarity, simplicity, and ambiguity by scoring each area in a special point scale. The experts suggested minor changes in wording but structure and clarity were reported. Following translation and validation process, a pilot study was conducted on 41 nurses (not included in main study) to measure the reliability and to determine the internal consistency of items, using the Cronbach’s alpha coefficient. An alpha value *α* > 0.7 indicates reliability of the items [23, 24]. The results revealed an alpha of 0.911 for QNWL, 0.758 for ATS, and 0.825 for OCQ.

### Statistical analysis

The data analysis was undertaken using the structural equation modeling software named Smart PLS (version 3). The software uses partial least squares (PLS) analysis for testing the study hypotheses. It was appraised based on the significance of the path coefficients and the squared multiple correlation (*R*^2^) values through focusing on the relationships between the dependent variable (turnover intention) and the independent variables (the quality of work life and organizational commitment). Descriptive statistics were applied to analyze the nurses’ characteristics. Frequency and percentage were determined by the Statistical Packages Software Services IBM SPSS version 24.

## Results

### Characteristics of respondents

The majority of participants were females (93%), had a diploma degree (95.6%), aged average approximately 31, and had less than 6 years of experience (85%). About half of nurses were married (53%) and had no children (62.3%). Most of the nurse’s ethnicity was Malay (94%), and 47.2% of respondents had income between RM2001 and RM3000. Table 1 shows the characteristics of respondents.

**Table 1:** Characteristics of nurse respondents (*n* = 430)

Demographic data		*n*	(%)
Nurses age	21–30	331	77
	31–40	92	21.4
	≥ 41	7	1.6
Gender	Male	30	7
	Female	400	93
Marital status	Not married	197	45.8
	Married	228	53
	Divorced	2	0.5
	Windowed	3	0.7
Had children	Yes	171	37.7
	No	259	62.3
No. of children	0	268	62.3
	≤ 2	126	29.3
	≥ 3	36	8.3
Ethnicity	Malaysia	404	94
	Chines	7	1.6
	India	13	3
	Other	6	1.4
Educational level	Diploma	411	95.6
	Bachelor	4	0.9
	Master	1	0.2
	Other	14	3.3
No. of years of experience	< 6 years	365	85
	≥ 6 years	65	15
Wards/units	Medical	147	34.2
	Surgical	117	27.2
	Special unit	166	38.6
Income	Less 2000	98	22.8
	2001–3000	204	47.4
	3001–4000	108	25.1
	More than 4000	20	4.7
Range of age (years)	21–51		
Average age (years)	31		

### The findings of the structural equation model Smart PLS

**Fig. 2:**
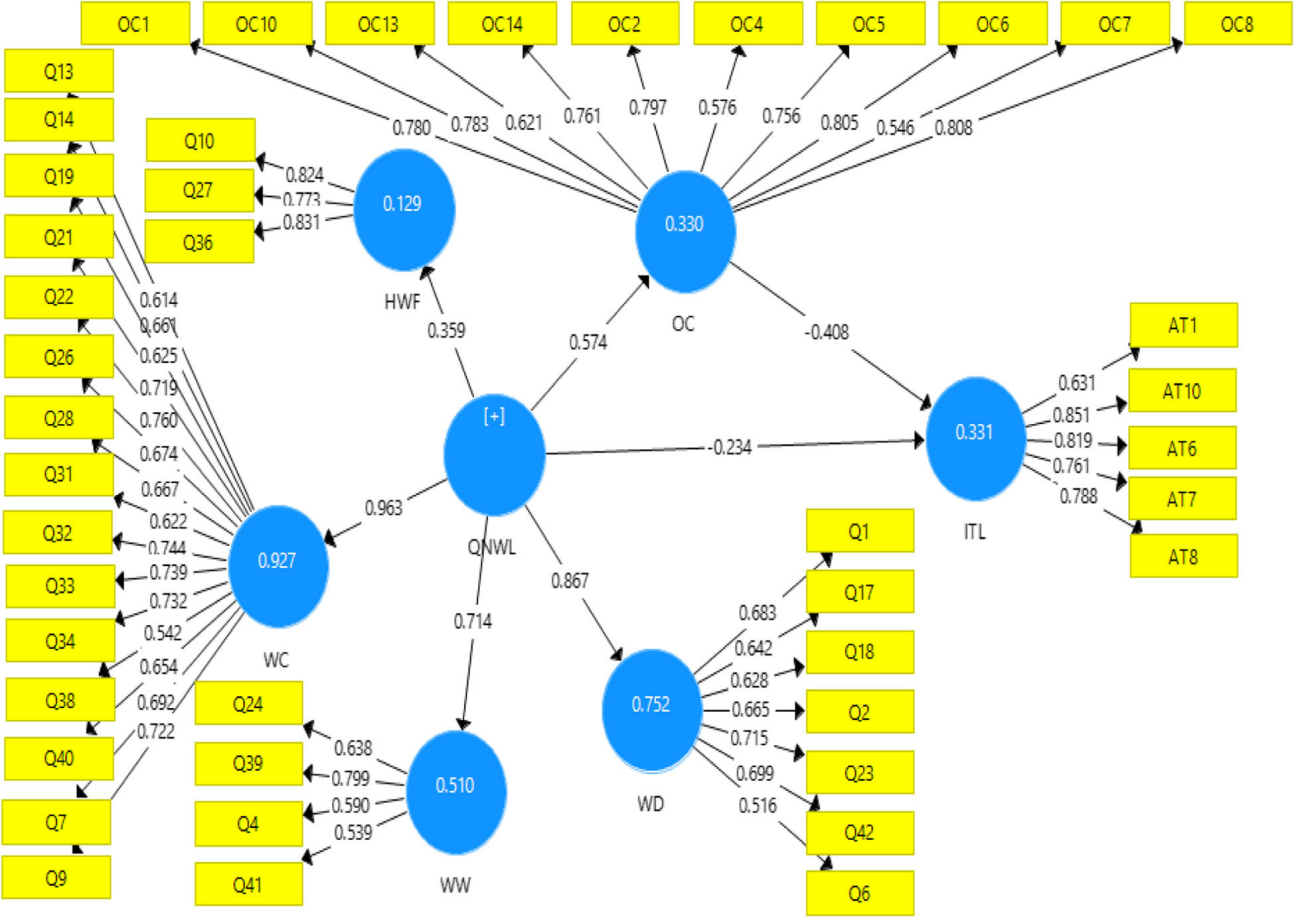
Structural model with mediators

**Fig. 3:**
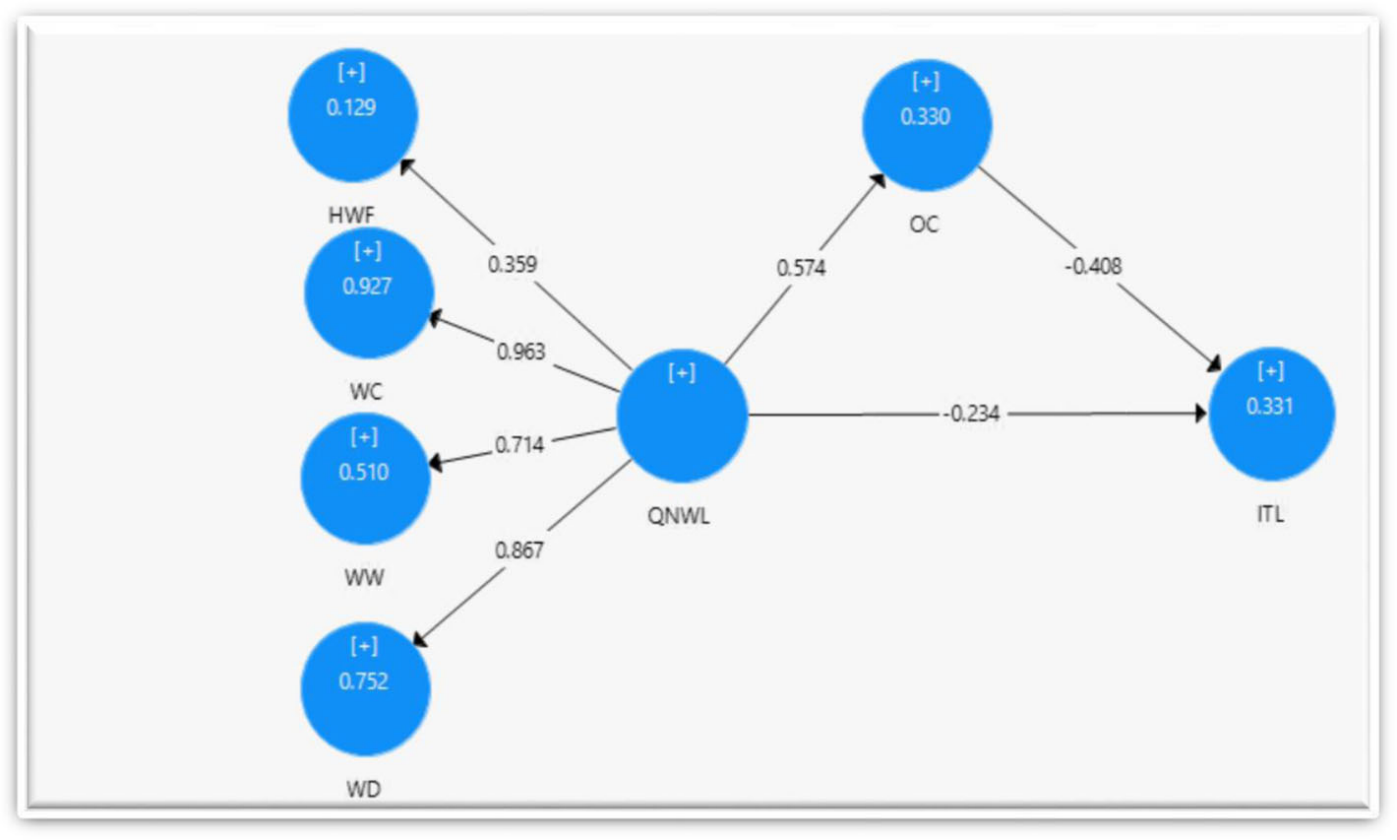
Structural model with mediator variables

The model is portrayed as a good fit with the data, as proven by the squared multiple correlation (*R*^2^) values of the dependent variables, TI at *R*^2^ = 0.331 and OC at *R*^2^ = 0.330, as shown in Figs. 2 and 3. Thus, the four latent variables of quality of work life factors (home/work life, work design, work context, work world) explain 33% of the variance in the organizational commitment’s mean. Meanwhile, the quality of nursing work life factors and organizational commitment explain 33.1% of registered nurses turnover intention in teaching hospitals. The loadings in this study ranged between 0.5 and 0.963, except the loadings of HWF (home/work life) which were at 0.359 and AVE at 0.586. The discriminate validity of the construct was at 0.810. On the other hand, the indicators are important for measuring the variables, hence cannot be removed. Thus, HWF remained in the model (Figs. 2 and 3).

Table 2 showed the influence of quality of work life (QWL) on turnover intention (TI) at a significant level (*P* value at 0.001), which means H1 is accepted. Moreover, the path coefficient β was − 0.234, indicating a negative relationship. One unit increase in the QWL equates to a decrease 0.0234 in the level of TI. Besides, Table 2 shows the significant effect of the QWL and OC at *P* value < 0.001, supporting H2 (QWL ➔ OC). Furthermore, the path coefficient β was 0.574, indicating a positive relationship with a large effect size. The results indicate a one-unit increase in the QWL will increase 0.574 unit of the level of OC. Finally, Table 2 indicates that there is a significant effect between OC and TI (< 0.001) that supports H3 (OC ➔ ITL). The path coefficient β was − 0.408, indicating a negative relationship. An increase of one unit in OC will lead to a decrease of 0.408 unit of the level of TI.

**Table 2:** Summary of structural model assessment of direct hypotheses

Hypotheses	Relation	Original sample (β)	*T* value	*P* value	Result
H1	QWL→QWL	− 0.0234	4.629	0.000	Accepted
H2	QWL→OC	0.574	14.181	0.000	Accepted
H3	OC→IT	− 0.408	7.685	0.000	Accepted

*Significant at bootstrapping *P* < 0.05

**Significant at bootstrapping *P* < 0.01

This study conducted a mediation analysis by applying SEM using partial least squares (PLS) to detect and estimate the mediating effect of organizational commitment (OC). The mediation test was based on the PLS bootstrapping approach. Therefore, the hypotheses were tested using PLS technique. The mediating impact was determined by means of a bootstrapping analysis, in tandem with the formulated hypotheses [25].

Table 3 shows that organizational commitment (OC) mediates the link between the quality of work life (QWL) and turnover intention (TI) among nurses in teaching hospitals, as an endogenous variable. This is because the T-statistics value for the hypothesis was 6.787 (more than 1.960) and the *P* value was less than 0.001. A path relationship in a SEM model is significant at *P* < 0.05 when the T-statistics value is > 1.96 [25]. Table 3 indicates a significant relation, QWL impacts TI and TI impacts OC with a β coefficient of − 0.234. More precisely, the direct effect of the relationship between QWL and TI was significant (*β* = − 0.228; *t* = 4.494).

**Table 3:** Summary of path coefficients and hypothesis testing (mediating results)

Hypothesis	Relation	Original sample (β)	Sample mean	*T* value	*P* value	Result
Direct effect	QWL→IT	− 0.228	0.051	4.494	0.000**	Accepted
Indirect effect	QWL→OC→IT	− 0.234	− 0.240	6.782	0.000**	Accepted
Total effect	QWL→IT	− 0.462	− 0.468	11.004	0.000**	Accepted

*Significant at bootstrapping *P* < 0.05

**Significant at bootstrapping *P* < 0.01

The mediating effect of QWL➔ TI is the multiplication of the regression weight of QWL➔ OC and the regression weight of OC ➔ TI, which is (0.574 × − 0.408) or − 0.234. It means one unit of increases in the mediator OC reduced 0.234 unit of the relationship between QWL and TI. The total effect in a mediation model is the total of the direct and mediating effect [25]. The total effect of QWL ➔ TI is described by the total influence of change effect of the relationship between QWL and TI with the occurrence of the mediating effect of OC. The total effect is *β* = − 0.462 [calculated by the total effect (*β* = − 0.462) = direct effect (*β* = − 0.228) + mediation effect (*β* = − 0.234)]. The result shows that the OC reduces the effect of the relation between QWL and TI from *β* = − 0.228 to *β* = − 0.462. It means organizational commitment has a negative partial mediating effect between work life quality of nurses and intention of turnover in teaching hospitals where the OC further reduced their intention. Table 3 presents the path coefficients for hypotheses testing.

## Discussion

The test of the first hypothesis displayed that there is a significant and reverse correlation between the quality of work life (QWL) and turnover intention (TI). The findings explained that work design, work context, work world, and home/work life have an impact on turnover intention. That means workload, work environment, home/work balance, and social interaction among Malaysian nurses influence turnover intention. Therefore, QWL should be approached properly to avoid actual turnover of nurses. These findings were similar to previous literature and provide evidence that the perception of QWL is negatively related to intention to leave their organization [8, 9, 26, 27].

The second hypothesis proved a significant and positive correlation between QWL and organizational commitment (OC). Organizations must put emphasis on QWL and its dimensions to retain a highly committed workforce. These results were consistent with the study by Eren and Hisar and Zhao et al., who both mentioned that the effect of QWL is positive on commitment [13, 15]. The results confirmed our study hypothesis that better quality work life means emotional attachment which helps to decrease turnover.

Additionally, the third hypothesis showed a significant and negative correlation between OC and TI. Similar results were reported by Ahmed et al. The organizational commitment had a statistically significant reverse correlation with the nurses’ intention of turnover their profession [16]. Mothoa and Lee et al. mentioned that organizational commitment is a statistically significant predictor of intention for turnover (*P* < .05) [17, 28]. In this study, it is further confirmed that commitment to organization has the potential to minimize intention of nurses to take decision for turnover. The results indicate that highly committed nurses are more loyal to the organization, hence staying with the organization.

Finally, the fourth hypothesis showed that organizational commitment partially mediated the relation between QWL and TI. The finding is compatible with the finding of a study conducted on faculty members in Saudi Arabia, as it was also found that the organizational commitment partially mediates between emotional exhaustion and intention of turnover [29]. Another study by Kamel on the faculty members in King Saud University proved that effective commitment has strong mediating effects on the relation between QWL and turnover intention [30]. Also, a study by Momani on 200 working Jordanian women revealed the link between the work-life balance and intention of turnover was mediating by effective commitment completely, whereas the normative mediates partially [31]. The results represented in this study show that QWL ensures greater organizational commitment to reduce nurses’ turnover intention. QWL and organizational commitment significantly reduce nurses’ turnover intention in the organization. Hence, such relationships warrant managers and decision makers to develop nurses’ retention strategy(s) in the organization.

## Conclusion

The present study concludes that the growing work life quality, one that reflects in the formal organizational policies and procedures in the hospitals, will improve nurses’ perception on the link between work life quality, commitment to organization, and intention for turnover. The findings confirm the importance of work life quality for nurses in promoting their obligation and retention in their organization. The organization should look into the nurses’ needs to improve work life quality for nurses, which will then improve commitment and decrease turnover intention. The commitment for organization has important impact on nurse’s quality work life and turnover intention, so more attention to its level is recommended.

## Data Availability

The datasets used and/or analyzed during the current study are available from the first author under the supervision of the corresponding author on reasonable request.

## Abbreviations

AVE: Average variance extracted
HWF: Homework family
OC: Organizational commitment
PLS: Partial least squares
QWL: Quality of work life
QNWL: Quality of nursing work life
*R*^2^: Squared multiple correlations
SEM: Structural equation model
SPSS: Statistical Packages Software Services
TI: Turnover intention
